# Small Extracellular Vesicles Derived From MSCs Have Immunomodulatory Effects to Enhance Delivery of ASO-210 for Psoriasis Treatment

**DOI:** 10.3389/fcell.2022.842813

**Published:** 2022-03-10

**Authors:** Weixian Zhang, Jingxiong Lin, Peilin Shi, Dandan Su, Xiaoli Cheng, Wenkai Yi, Jian Yan, Hongbo Chen, Fang Cheng

**Affiliations:** ^1^ School of Pharmaceutical Sciences (Shenzhen), Shenzhen Campus of Sun Yat-sen University, Shenzhen, China; ^2^ Department of Pharmacy, Shenzhen Baoan Maternal and Child Health Hospital, Shenzhen, China; ^3^ Department of Biomedical Sciences, City University of Hong Kong, Hong Kong SAR, China

**Keywords:** small extracellular vesicles, human umbilical cord MSCs, antisense oligonucleotides-210, psoriasis, MSC-sEVs

## Abstract

Mesenchymal stem cells (MSCs) have been increasingly used for treating autoimmune diseases due to their immune modulation functions, but inefficient homing to the target tissue and safety issues limits their wide application. Recently, increasing studies demonstrate small extracellular vesicles (sEVs) as key mediators of MSCs to exert their immunomodulatory effects. In this study, we found that sEVs derived from human umbilical cord MSCs stimulated by IFN-γ (IFNγ-sEVs) inhibited proliferation and activation of peripheral blood mononuclear cells and T cells *in vitro*. Furthermore, we confirmed that IFNγ-sEVs reduced psoriasis symptoms including thickness, erythema, and scales of skin lesions; exhausted Th17 cells, increased Th2 cells; and reduced enrichment of inflammatory cytokines such as IL-17A, IFN-γ, IL-6, and TNF-α in both spleen and skin lesions *in vivo*. Importantly, IFNγ-sEVs significantly improved the delivery efficiency and stability of ASO-210, the antisense oligonucleotides of miR-210 block the immune imbalance and subsequent psoriasis development. Our results reveal MSC-sEVs as promising cell-free therapeutic agents and ideal delivery vehicles of antisense oligonucleotides for psoriasis treatment.

## Introduction

Psoriasis is a chronic, inflammatory cutaneous disease. Patients with psoriasis suffer from both physical and mental problems. So far, the pathogenesis of psoriasis is complex and not fully elucidated. Cross-talk between excessive activated immune cells and hyperproliferative keratinocytes in the skin is thought to be central to the pathogenesis of psoriasis (Nestle and Ba Rker, 2009; Chiricozzi et al., 2011; Alwan and Nestle, 2015; Armstrong and Read, 2020). Adaptive immune cells such as T helper (Th) cells (Lowes et al., 2008; Kagami, 2013; Hartwig et al., 2018) and innate immune cells including dendritic cells (DCs), natural killer T (NKT) cells, and macrophages, secreting various cytokines, are thought to be the key players in the pathogenesis of psoriasis (Frischknecht et al., 2019; Egeberg et al., 2020). For example, the activated dendritic cells secrete IL-23 and then mediate the activation of Th17 cells and induce the cytokines release of IL17 A/F, TNF-α, and IL-22 (Nickoloff, 2007; Becher and Pantelyushin, 2012). These cytokines lead to keratinocyte over-proliferation, increased expression of angiogenic mediators and endothelial adhesion molecules, and infiltration of immune cells into skin lesions further (Gupta et al., 2021).

The current therapeutic options for psoriasis include topical therapies, phototherapies, and systemic therapies (Armstrong and Read, 2020). Topical agents such as salicylic acid, retinoids, and corticosteroids are preferred for patients to avoid unwanted systemic effects (Mason et al., 2009; Leonardi et al., 2015; Gold et al., 2018). Therapeutic advancements for moderate-to-severe plaque psoriasis include biologics that inhibit TNF-α, IL-12/23, IL-17A/F, and IL-23, and an oral phosphodiesterase 4 inhibitor. Although these agents such as monoclonal antibodies and corticosteroids have substantially improved the effectiveness of psoriasis treatment, the long-term use may increase the risks of serious infection, cancer, and other serious disorders (Bruner et al., 2003). Therefore, it is important to develop more effective and safer therapeutic strategies for patients with moderate-to-severe psoriasis.

In recent years, mesenchymal stem cells (MSCs) such as adipose tissue-derived MSCs (AT-MSCs) and umbilical cord-derived MSCs (UC-MSCs) are being tested in several clinical trials for psoriasis treatment (ClinicalTrials: NCT02491658, NCT03424629, CNCT03265613, NCT04275024, NCT04785027, and NCT03765957). However, the major concern with the use of MSCs is related to their survival and multilineage differentiation potential *in vivo*, which might result in the formation of the wrong cell types and could even induce the tumor formation, so safety issues need to be taken into account when MSCs are used in humans. In addition, MSCs by systemic transplantation are quickly entrapped in the lung vasculature bed due to their big size, leading to a small number of MSCs (<1%) reaching to the target sites (Singer and Caplan, 2011; Galipeau and Sensébé, 2018). Increasing studies show that the therapeutic effect of MSCs, especially immunomodulatory effects, is mediated by small extracellular vesicles (sEVs) (Riazifar et al., 2019; Witwer et al., 2019; Song et al., 2020). Accumulating evidence suggests that sEVs display immunoregulatory functions similar to the parent MSC. These sEVs can be also modified or induced by various cytokines to enhance inherent immunomodulatory potential. These MSC-sEVs may be more effective than MSCs themselves, since sEVs can be produced and purified massively, administered systematically with minimal toxicity, and reach the target tissues and cells efficiently (Heldring et al., 2015; Mardpour et al., 2019; Pu et al., 2020). These all indicated that MSC-sEVs could be developed as cell-free therapeutics for psoriasis.

MicroRNAs (miRNAs) are endogenous small non-coding RNA molecules comprising (18–25) nucleotides that can negatively regulate gene expression via promoting target mRNA degradation (Bartel, 2009). A previous study reported that miRNAs 210, comprising 22 nucleotides, were highly expressed in both psoriasis patients and psoriasis-like mouse models. Increased expression of miRNAs enhanced Th1 and Th17 cells in peripheral blood and skin lesions of both psoriasis patients and mouse models, which can exacerbate inflammation in skin lesions of psoriasis. Antisense oligonucleotides (ASOs) of miR-210 (ASO-210) were demonstrated to alleviate psoriasis by blocking the immune imbalance and the development of psoriasis-like inflammation (Wu et al., 2018). However, instability *in vivo* and efficient delivery to the target cell of ASO-210 is a major challenge for its clinical application. Therefore, improving its stability and delivery efficiency is desired. Interestingly, with the small size, high stability, lower cytotoxicity and immunogenicity, and target specificity, sEVs have been identified as natural nanocarriers for nucleotide drugs (Kim et al., 2020; Zhang et al., 2020; Gagliardi and Ashizawa, 2021; Yang et al., 2021). Collectively, the combination therapy of MSC-sEVs carrying ASO-210 may be considered as a promising strategy to psoriasis patients.

In our study, we found that sEVs derived from human umbilical cord tissue-derived MSCs (huc-MSCs) stimulated by IFN-γ (IFNγ-sEVs) can inhibit PBMC and T-cell proliferation and activation *in vitro*. IFNγ-sEVs accumulated in the injured skin and alleviated psoriasis symptoms, reduced dermal thickness, and inhibited proliferation of keratinocytes. In addition, we observed a decreased number of CD4^+^IL-17A^+^ T cells (Th17), an increased number of CD4^+^IL-4^+^ T cells (Th2), and significantly reduced inflammatory cytokine mRNA expression such as *Il17a*, *Ifng*, *Il6*, and *Tnfa* in both spleen and skin lesions of IFNγ-sEVs-treated mice. In order to improve the delivery efficiency and stability, we encapsulated ASO-210 into IFNγ-sEVs through electroporation. Compared with ASO-210 just mixed with IFNγ-sEVs in the buffer (ASO-210&IFNγ-sEVs), ASO-210 loaded into EVs (ASO-210@IFNγ-sEVs) presents better symptom alleviation of psoriasis. Together, our results demonstrate MSC-sEVs as efficient immunosuppressive agents and a promising tool to enhance the delivery of ASO-210 in psoriasis treatment.

## Materials and Methods

### Isolation of Huc-MSCs

Human umbilical cord tissue-derived MSCs (huc-MSCs) were isolated from discarded umbilical cords and prepared following an established protocol. All procedures were conducted under aseptic, standardized conditions. Fresh umbilical cord specimens were obtained from healthy term neonates and stored in PBS at 4°C. In the ultra-clean table, the cords were cut into 3 to 4 cm pieces and were rinsed with 5% penicillin–streptomycin PBS buffer to remove the cord blood and other impurities. The outer membrane and the three blood vessels in cords were separated in turn, and Wharton jelly was obtained from the viscous substance. Wharton jelly was cut into 1-mm^3^ pieces and was dispersedly transferred into a Petri dish with DMEM-F12 containing 10% fetal bovine serum, 1% penicillin–streptomycin, and 10 ng/ml bFGF at 37°C in a cell incubator with 5% CO_2_. In the primary culture, half of the medium was changed 4 days later. Then, the culture medium was refreshed every 3 days. Generally, fibroblast-like cells can be observed to crawl out after 14 days. When they reached 70–80% confluence, the cells were subcultured to a new Petri dish for further expansion.

### Cell Culture and sEV Isolation

DMEM-F12 supplement with 10% fetal bovine serum, 5% penicillin–streptomycin, and 10 ng/ml bFGF was used to culture huc-MSC. To obtain more and purer sEVs, huc-MSC with passages two to three was transferred to the DMEM-F12 medium supplemented with 0.5% fetal bovine serum and 10 ng/ml IFN-γ. After 48 h, the culture medium was collected and then centrifuged at 1,000×g for 10 min, 4,000×g for 20 min, and 10,000×g for 40 min successively to remove cells, cells debris, and microvesicles. Subsequently, huc-MSC-sEVs were pelleted by ultracentrifugation at 100,000×g for 70 min and then resuspended in PBS. Moreover, all centrifugation processes were performed at 4°C.

### Characterization of sEVs

The size distribution and zeta potential of sEVs were measured using a NanoBrook 90 Plus PALS (Brookhaven) instrument. The morphology of sEVs was detected using a transmission electron microscope (TEM).

### Western Blotting

Cell and sEV lysates were separated using the SDS-PAGE and were then transferred to polyvinylidene fluoride membranes (Millipore, Darmstadt, Germany). Then the membrane was sealed with 5% non-fat milk for 1.5 h at room temperature and incubated with the desired primary antibodies (CD63, CD9, Alix, TSG101, STAT6, LYN, and β-actin) overnight at 4°C. Post-incubation with HRP-conjugated secondary antibodies was performed for 1.5 h at room temperature, and then detection was carried out using an enhanced chemiluminescence reagent (ECL) (Protein Tech, China**).**

### RNA Isolation and RT-qPCR

Total RNAs from cells, skin tissue, and spleen tissues were extracted using the TRIzol reagent according to the manufacturer’s protocol. The skin tissue and spleen were added to the TRIzol reagent and grounded. The RNA concentration was measured using a NanoDrop One (Thermo Fisher Scientific). Then, according to the manufacturer’s manual, the RNAs were reversely transcribed into cDNA using a QuantiTect RT Kit (Qiagen, Valencia, CA, United States), according to the manufacturer’s protocols. All the primers used in this study are listed in Table 1.

**TABLE 1:** Sequences of the qPCR primers.

Gene	Forward primer sequence 5’→3′	Reverse primer sequence 5’→3′
H-*IDO1*	TCT​CAT​TTC​GTG​ATG​GAG​ACT​GC	GTG​TCC​CGT​TCT​TGC​ATT​TGC
H-*PEG2*	TCC​TAA​CCC​TTT​TGT​CGC​CTG	CGC​TTC​CCA​GAG​GAT​CTG​C
H-*IL10*	TCA​AGG​CGC​ATG​TGA​ACT​CC	GAT​GTC​AAA​CTC​ACT​CAT​GGC​T
H-*GADPH*	GGA​GCG​AGA​TCC​CTC​CAA​AAT	GGC​TGT​TGT​CAT​ACT​TCT​CAT​GG
H-*STAT6*	GGA​GCA​CCA​TCT​TGC​AAC​AC	GTG​GCG​GAA​CTG​TTC​CAT​AA
H-*LYN*	ATG​TGA​GAG​ATC​CAA​CGT​CCA​A	AAA​AGC​TGC​CTT​TCT​GCG​TC
M-*Il17a*	ATG​CTG​TTG​CTG​CTG​CTG​AG	GGA​AGT​CCT​TGG​CCT​CAG​TG
M-*Tnfa*	CAT​CTT​CTC​AAA​ATT​CGA​GTG​ACA​A	TGG​GAG​TAG​ACA​AGG​TAC​AAC​CC
M-*Ifng*	AGA​CAA​TCA​GGC​CAT​CAG​CA	CAA​CAG​CTG​GTG​GAC​CAC​TC
M-*Il6*	GAG​GAT​ACC​ACT​CCC​AAC​AGA​CC	AAG​TGC​ATC​ATC​GTT​GTT​CAT​ACA
M-*Gadph*	AGG​TCG​GTG​TGA​ACG​GAT​TTG	TGT​AGA​CCA​TGT​AGT​TGA​GGT​CA

### Loading of ASO-210 Into sEVs

All the antisense miRNA-210 oligonucleotides were designed and purchased from GenePharma. The sequence of ASO-210 is 3′ GAC​ACG​CAC​ACU​GUC​GCC​GAC​U 5’. To encapsulate ASO-210 into sEVs, 1 mg (protein weight) purified IFNγ-sEVs and 100 nmol ASO-210 were gently mixed in 2 ml electroporation buffer (1.15 mM potassium phosphate pH 7.2, 25 mM potassium chloride) at 4°C. The mixtures were subjected to electroporation at 400V and 150 μF in to 0.4-cm electroporation cuvettes using a MicroPulser Electro-operator (Bio-Rad, United States). After electroporation, the mixtures were incubated at room temperature for 30 min for the membrane recovery. Unloaded ASO-210 was removed by ultracentrifugation at 100,000×g for 70 min. Then supernatants were removed, and the pellets were resuspended in 2 ml PBS. At last, the sample was diluted 50 times with PBS for test concentration. FAM-labeled ASO-210 was electroporated with sEVs as described before. The amount of FAM-ASO-210 was quantified using a fluorometer with excitation at 490 nm and emission at 520 nm. The loading efficiency was calculated as follows: Loading efficiency (%) = (Amount of AMO-210 after electroporation)/(Total amount of ASO-210 before electroporation) ×100.

### The uptake of sEVs and ASO-210@sEVs in Jurkat T and HaCaT cells

Jurkat cells and HaCaT cells were, respectively, seeded in a confocal plate at a density of 10^6^ cells and cultured for 24 h. Jurkat T cells and HaCaT cell membranes were pre-stained with WGA 350 for 10 min at 37°C. The sEVs and ASO-210@sEVs were stained with DiD for 10 min at 37°C. Then, the DiD-labeled sEVs were added to both Jurkat cells and HaCaT cells for 30–60 min. The cells were observed using a confocal microscope (Zeiss, LSM880).

### Isolation and Proliferation of PBMCs and T Cells From Human Peripheral Blood

PBMCs were harvested from healthy donor’s blood and isolated by Ficoll gradients. Fresh anticoagulated whole blood was added onto the half volume of Ficoll-Paque PLUS (Sigma-Aldrich) in a centrifuge tube, and centrifuged at 1500 rpm for 40 min. The PBMC interface layer was gently collected and washed twice with filter-sterilized PBS by centrifugation at 1000 rpm for 10 min. Next, CD3^+^ T cells were separated from freshly PBMCs by using a MojoSort Human CD3^+^ T-cell Isolation Kit (Biolegend, United States), cultured in CD3-coated plates (clone OKT3; Biolegend), and then cultured in medium (RPMI1640 with 10% FBS, penicillin/streptomycin, and 2 ng/ml IL-2).

## CFSE Staining

Freshly isolated PBMCs and CD3^+^ T cells were labeled with a CFSE working solution (5 μM) using a CFSE division tracker kit (BioLegend, United States) for 20 min at 37°C and were quenched staining with the culture medium. Then PBMCs and CD3^+^ T cells were seeded in a 24-well plate coated with 10 ug/ml anti-CD3 (clone OKT3; Biolegend) and cultured in a medium (RPMI1640 with 10% FBS, penicillin/streptomycin, and 2 ng/ml IL-2). At days 0 and 5, respectively, PBMCs and T cells were collected and subjected to FACS (Cytoflex, Beckman, United States) using Cell Quest software (CytExpert, United States).

### IMQ-Induced Psoriasis Mouse Model

In total, 6- to 8-week-old male BALB/c mice were purchased from the Laboratory Animal Center of Sun Yat-Sen University and kept in a pathogen-free facility. To induce psoriasis, the mice were treated with a daily topical dose of 62.5 mg of imiquimod (IMQ) cream on the shaved back for 7 consecutive days.

### Distribution of MSC-sEVs *In Vivo*

DiD-labeled native sEVs (150 μg) and IFNγ-sEVs (150 μg) were intradermally injected into the BALB/c mice. After 12 h, accumulation of sEVs in various organs was observed using a Night OWL imaging system (LB983).

### PASI of Skin Inflammation in IMQ-Induced Psoriasis Mice

The severity of back skin inflammation in the IMQ-induced psoriasis-like mouse model was scored by an objective scoring system, which was based on clinical PASI for psoriasis patients. Thickening, scaling, and erythema were scored independently on a scale from 0 to 4: 0, none; 1, slight; 2, moderate; 3, marked; and 4, very marked. The total score was obtained by accumulating the three index scores (scores 0–12).

### H&E Staining and Immunohistochemistry Staining

Skin tissue samples from the psoriasis site were collected and fixed with 3 ml 4% paraformaldehyde for 24 h before transferring into 70% ethanol. Then, the samples were embedded with paraffin and cut into 4-μm-thick sections. The sections were stained with hematoxylin and eosin (H&E) following the standard procedure. The inflammatory cell infiltration condition was observed using a light microscope with ×100 magnification. Immunohistochemistry was performed to confirm the counts of Ki67^+^, CD3^+^, IL-6^+^, and TNF-α^+^ cells in psoriasis skin lesions. In brief, all formalin-fixed paraffin-embedded tissue samples were subjected to deparaffinization, rehydration, and heat-induced epitope retrieval and were subsequently incubated with Ki67^+^, CD3^+^, IL-6^+^, and TNF-α^+^ antibodies (1:50 in 1.5% BSA) overnight. Afterward, secondary antibodies were incubated at room temperature in the dark for 1 h. After washing with PBS, Ki67^+^, CD3^+^, IL-6^+^, and TNF-α^+^ cells were observed by microscopy.

### Cell Preparation for Flow Cytometry

Spleen and lymph nodes collected from mice were immersed in PBS in 1.5-ml centrifuge tubes and kept in ice. For cell isolation, the spleen and lymph nodes were repeatedly grounded, washed with PBS, and filtered through a 100-μm filter. Cells were collected by centrifuging at 1,500 rpm for 5 min, before resuspending in cell staining buffer (Biolegend, United States), and were incubated with the FITC anti-mouse CD3 antibody (Biolegend, catalog 317306) and APC anti-mouse CD4 antibody on ice for 15 min in the dark. For intracellular staining, the cells were fixed in fixation buffer (Biolegend, United States) in the dark for 20 min at room temperature, permeabilized, and stained in the dark for 15 min at room temperature with Brilliant Violet 510™ anti-mouse IL-17A (Biolegend, catalog 504127), PerCP-Cy5.5 anti-mouse IFN-γ (Biolegend, catalog 505822), and Brilliant Violet 421™ anti-mouse IL-4 (Biolegend, catalog 504127). The cells were washed with PBS, centrifuged, and filtered through a 100-μm filter before analysis.

### Statistics

Data analysis was performed by GraphPad Prism version 7.0 software. Data are presented as mean ± SEM. We assessed data for normal distribution and similar variance between groups. Statistical significance (*p < 0.05, **p < 0.01, and ***p < 0.001) was assessed using 2-tailed unpaired Student’s t-test for comparisons between two groups and one-way analysis of variance (ANOVA) with relevant post hoc tests for multiple comparisons. When the data were not normally distributed or displayed unequal variances between two groups, we used the 2-tailed Mann–Whitney U test for statistical analysis.

## Results

### Preparation and Characterization of huc-MSC-Derived sEVs

**FIGURE 1:**
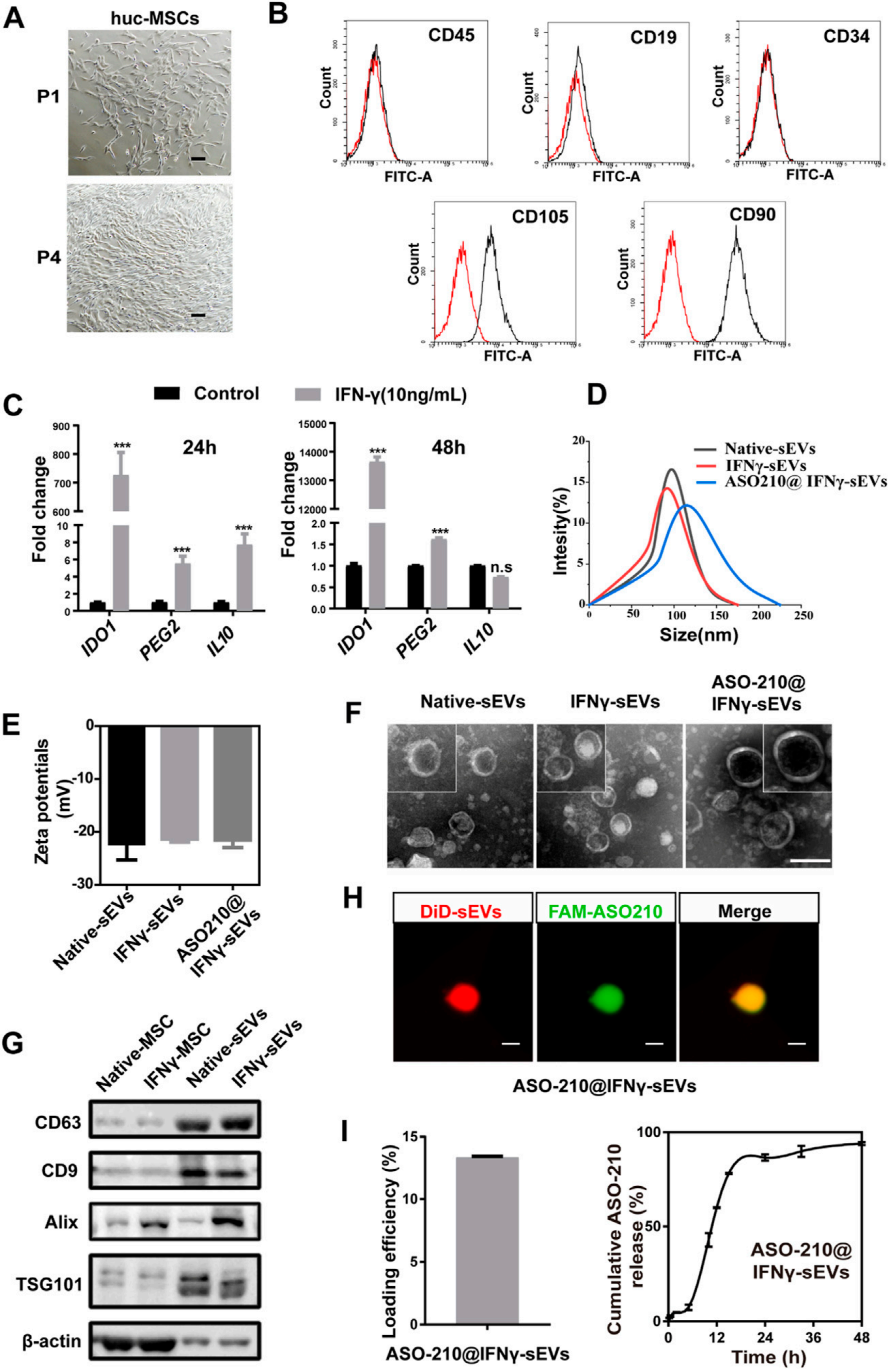
Preparation and characterization of huc-MSC-derived sEVs. **(A)** The spindle-shaped, fibroblast-like morphology of isolated huc-MSCs (P1 and P4). Scale bar: 100 μm. **(B)** Flow cytometry analysis of CD9, CD19, CD34, CD90, and CD105 expression on the huc-MSC surface. **(C)** qPCR of *IDO1*, *PEG2*, and *IL10* mRNA levels in huc-MSC stimulated with or without IFN-γ (n = 3). **(D,E)** The size distribution and zeta potential of native sEVs, IFNγ-sEVs, and ASO-210@ IFNγ-sEVs were measured by DLS (n = 3). **(F)** Representative TEM images of native sEVs, IFNγ-sEVs, and ASO-210@IFNγ-sEVs. Scale bar: 100 nm. **(G)** Western blotting of MSC-derived sEVs for CD9, CD63, TSG101, and Alix. **(H)** Representative confocal images of pre-stained DiD-sEVs (red) colocalized with FAM-labeled ASO-210 (green). Scale bar: 200 nm. Error bar, mean ± SD. n.s represents no significance, *p < 0.05, **p < 0.01, and ***p < 0.001. **(I)** ASO-210 loading efficiency and release efficiency of ASO-210@IFNγ-sEVs.

In order to test the potential of MSC-sEVs as the potential immunosuppressant drug to prevent the pathology of psoriasis, MSCs were isolated from fresh human umbilical cords (huc-MSCs) and expanded for three or four generations *in vitro* for sEV purification (Figure 1A). Flow cytometry analysis was used to confirm that the isolated huc-MSCs were positive for mesenchymal stem cell markers CD90, CD105, and negative for hematopoietic stem cell markers CD34, CD9, or CD45 (Figure 1B). IFN-γ, a potent multifunctional cytokine which is secreted primarily by activated NK cells and T cells, has recently been reported to promote immunosuppressive effects of MSCs (Riazifar et al., 2019). We found that the mRNA expression of a panel of immunosuppressive markers including *IDO1*, *PGE2*, and *IL10* was significantly increased in huc-MSCs with IFN-γ stimulation (Figure 1C **)**.

Next, we prepared and purified sEVs derived from native and IFN-γ-stimulated huc-MSCs by differential centrifugation. Dynamic light scattering (DLS) analysis showed that the particle size distribution is Gaussian, with a peak value of 91.86 and 97.06 nm, and the zeta potentials were -22.33 and -21.57 mV, respectively, indicating that the extracellular vesicles are well dispersed and stable (Figures 1D,E). When visualized under a TEM, the particles were spherically shaped with a typical bilayer membrane structure within the size range of sEVs in diameter (Figure 1F). Western blotting confirmed the presence of sEV markers, namely, CD63, CD9, TSG101, and Alix in isolated vesicles (Figure 1G).

ASO-210 has been reported to alleviate psoriasis by blocking the immune imbalance and inhibiting the development of psoriasis-like inflammation (Wu et al., 2018). However, instability and delivery inefficiency to target cells of ASO-210 hinder its clinical application. Therefore, we speculate that sEVs from primary huc-MSCs may be used as a targeted drug delivery vehicle to improve the therapeutic effects of ASO-210. In order to test the potential of IFNγ-sEVs as a synergistic, targeted drug delivery system of ASO-210 to effector cells, FAM-labeled ASO-210 was loaded into the MSC-derived IFNγ-sEVs by electroporation (ASO-210@IFNγ-sEVs), with an average loading efficiency of 13.35% and a release efficiency of 94.02% (Figure 1I). Similar to native and IFN-γ-stimulated sEVs, the average size of ASO-210@sEVs was 115 nm, and the mean zeta potential was -22.48 mV (Figures 1D,E), with membrane-bounded round shape within the size range of sEVs (Figure 1F). In addition, we also used confocal analysis to ensure that FAM-labeled ASO-210 is maintained in DiD-labeled sEVs, indicating that the ASO-210 was entrapped into sEVs (Figure 1H). To sum up, all the aforementioned experiments demonstrated the success of obtaining sEVs from primary huc-MSCs, which could carry ASO-210 for the following experiments.

### ASO210@IFNγ-sEVs Inhibit the Proliferation of PBMCs and T cells *In Vitro*.

**FIGURE 2:**
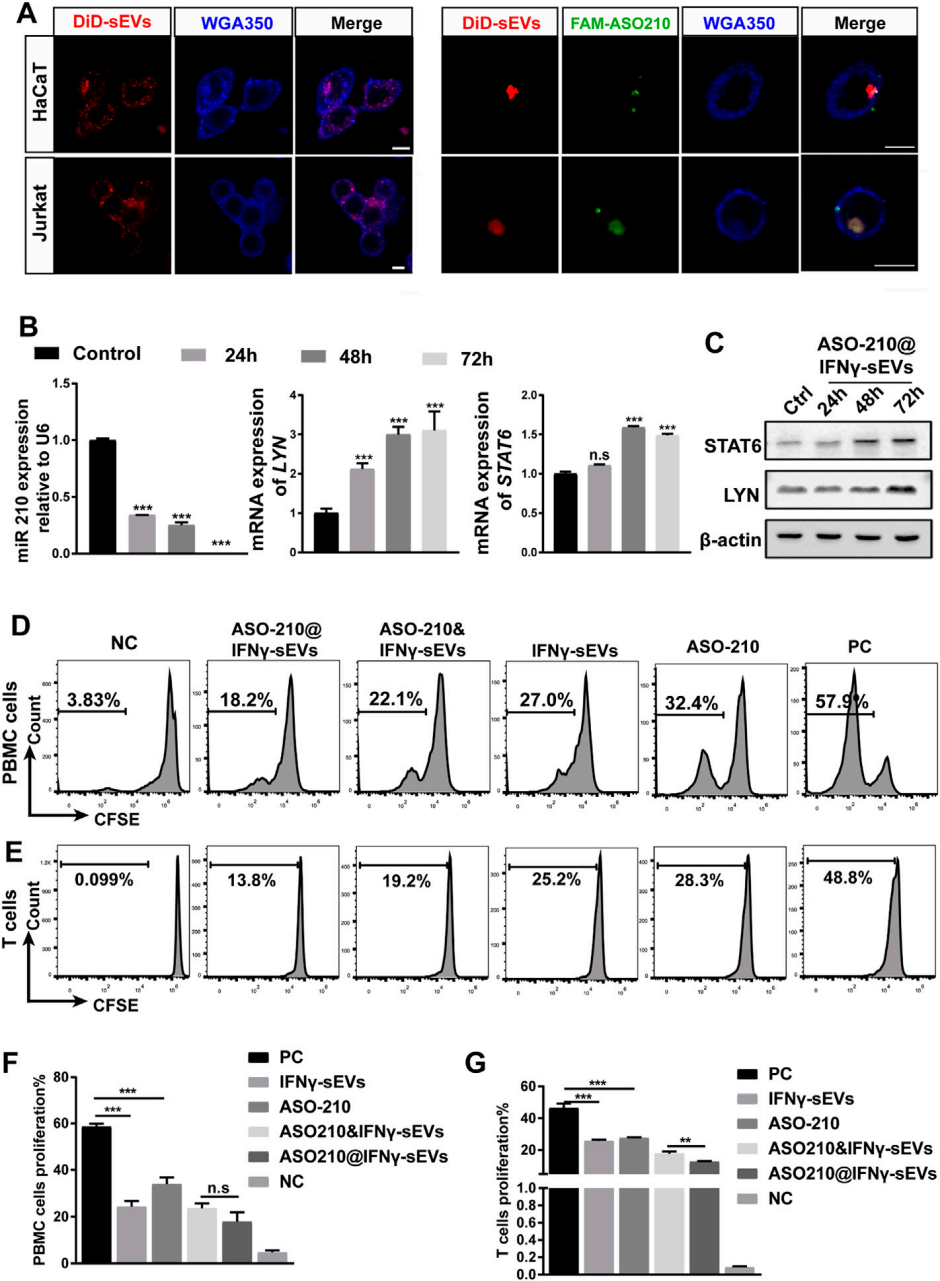
ASO-210@IFNγ-sEVs inhibit the proliferation of PBMCs and T cells *in vitro*. **(A)** Representative confocal images of HaCaT and Jurkat cells when incubated with IFNγ-sEVs and ASO-210@IFNγ-sEVs. Scale bar: 10 μm. **(B)** Representative quantitative analysis of the effect of ASO-210 at time points on the mRNA expression of miRNA-210, *STAT6*, and *LYN* in Jurkat T cells (n = 3). **(C)** Representative Western blot of ASO-210 at time point on the expression of STAT6 and LYN in Jurkat T cells. **(D,E)** Flow cytometry analysis of the proliferation of PBMC or T cells in groups that received different treatments (IFNγ-sEVs, ASO-210, ASO-210&IFNγ-sEVs, and ASO-210@ IFNγ-sEVs) for 5 days. The PC group stands for PBMC or T cells cultured for the same days without drugs. NC group stands for PBMC or T cells on day 0. **(F,G)** The corresponding quantitative analysis of PBMC or T cells from different treatment groups (n = 3). Error bar, mean ± SD. n.s represents no significance, *p < 0.05, **p < 0.01, ***p < 0.001.

Next, we determined whether IFNγ-sEVs can be used as a targeted drug delivery system to carry ASO-210 to effector T cells to enhance immune suppression. First, we examined the cellular uptake of IFNγ-sEVs and ASO210@IFNγ-sEVs in HaCaT keratinocytes and Jurkat T cells, two key target cell types of ASO-210 in psoriasis. The confocal images suggested that both IFNγ-sEVs and FAM-ASO210@IFNγ-sEVs could be used by both cell lines upon co-culture for 0.5 h (Figure 2A). A previous study has reported that ASO-210 can target miR-210 and thus activate the expression of STAT6 and LYN, two direct target genes of miR-210 (Wu et al., 2018). To investigate whether ASO210@IFNγ-sEVs can deliver ASO-210 into the cells to target the miR-210 pathway, Jurkat T cells were incubated with ASO-210@IFNγ-sEVs (1 nM ASO-210) for 0h, 24 h, 48 h, and 72 h. As expected, miR-210 expression in Jurkat T cells was dramatically reduced (Figure 2B), whereas both mRNA and protein levels of STAT6 and LYN increased significantly as the incubation time extended (Figures 2B,C). To investigate the biological role of IFNγ-sEVs and ASO-210 on the inhibition of T-cell activation, CFSE-labeled activated healthy human PBMCs or CD3^+^ T cells were treated with IFNγ-sEVs, ASO-210, ASO-210&IFNγ-sEVs, and ASO-210@IFNγ-sEVs for 5 days, respectively. The following flow cytometry analysis showed that IFNγ-sEVs, ASO-210, ASO-210&IFNγ-sEVs, and ASO-210@IFNγ-sEVs significantly inhibited the PBMCs proliferation by 28.28%, 18.58%, 29.98%, and 34.71%, respectively, suggesting that both IFNγ-sEV and ASO-210 presented immunosuppressive effects on activated PBMCs (Figure 2D). In addition, compared to ASO-210 mixed with IFNγ-sEVs (ASO-210&IFNγ-sEVs), ASO-210 loaded into IFNγ-sEVs (ASO-210@IFNγ-sEVs) greatly enhanced its immune inhibition function (Figures 2D,F). Furthermore, IFNγ-sEVs, ASO-210, ASO-210&IFNγ-sEVs, and ASO-210@IFNγ-sEVs have similar inhibition on proliferation of CD3^+^ T cells with PBMCs by 20.58%, 18.68%, 28.45%, and 33.58%, respectively (Figures 2E,G). This was supported by the corresponding quantitative analysis of CFSE results (Figures 2F,G).

### Intradermal Administration of IFNγ-sEVs Loaded With ASO-210 Ameliorates the Pathological Phenotype of IMQ-Induced Psoriasis-Like Mice

**FIGURE 3:**
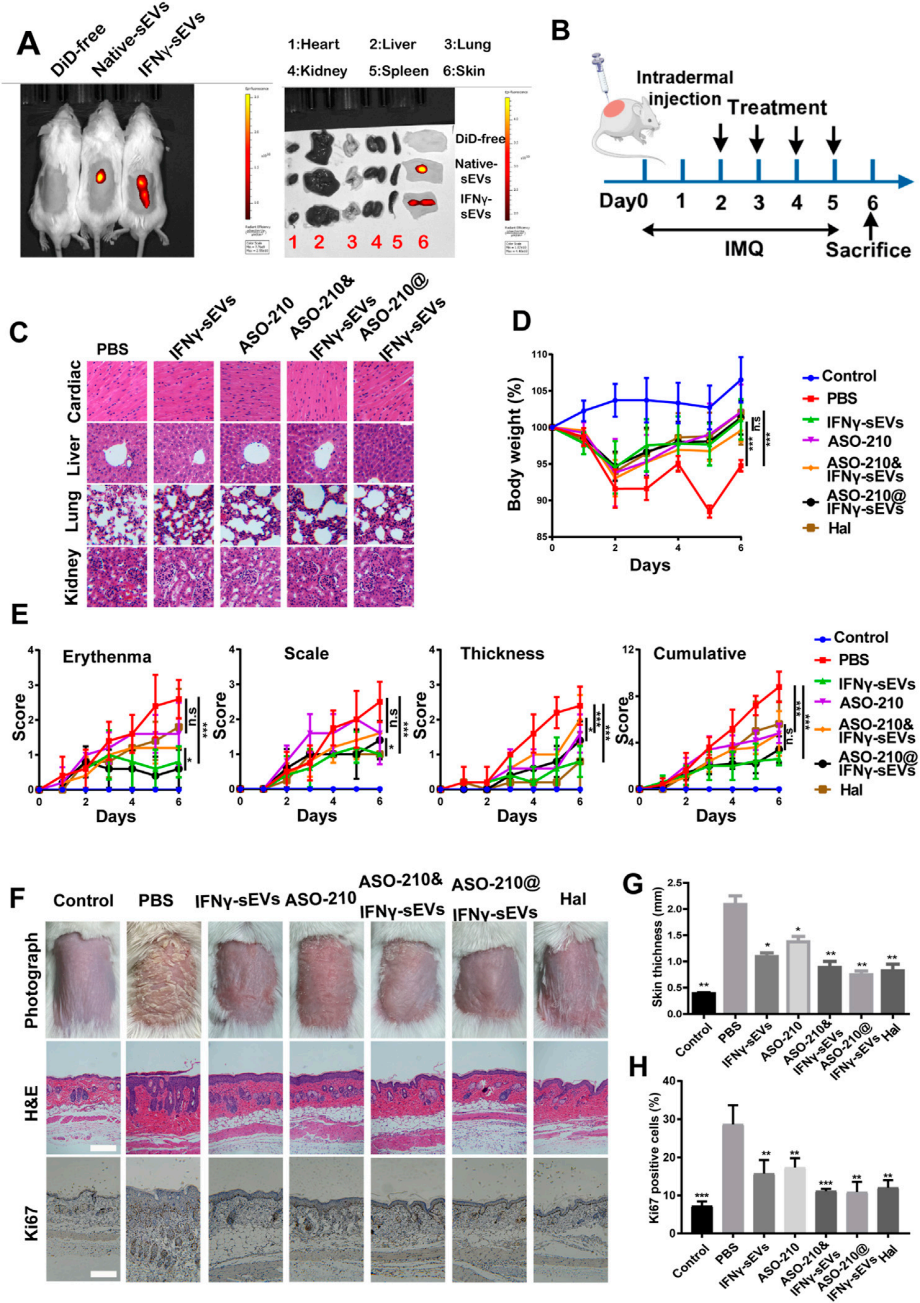
Intradermal administration of IFNγ-sEVs loading with ASO-210 ameliorates the pathological phenotype of IMQ-induced psoriasis-like mice. **(A)** *In vivo* distribution of native sEVs and IFNγ-sEVs in IMQ-induced psoriasis-like mice. **(B)** Schematic diagram for intradermal administration of IFNγ-sEVs, ASO-210, ASO-210&IFNγ-sEVs, and ASO-210@IFNγ-sEVs on day 2, 3, 4, and 5 during the application of IMQ in BALB/c mice. **(C)** Safety evaluation of IFN-γ and ASO-210 Scale bar: 40 μm. **(D)** The body weight of the mice in different groups (n = 5). **(E)** Thickness, erythema, scales, and accumulative score of different groups were recorded daily from day 0 to day 6 (n = 5). **(F)** Representative skin surface morphology, H&E staining and Ki67 immunohistochemistry of the skin lesions of mice in each group on day 7. Scale bar: 100 μm. **(G)** Skin thickness was measured using Image Pro Plus on the day 6, n = 4/5. Error bar, mean ± SD. n.s represents no significance, *p < 0.5, **p < 0.01, and ***p < 0.001. **(H)** Ki67^+^ cell percentage in psoriatic skin lesion was calculated by ImageJ on day 6, n = 4/5. Error bar, mean ± SD. n.s represents no significance, *p < 0.5, **p < 0.01, ***p < 0.001.

We next sought to investigate the therapeutic effect of IFNγ-sEVs and ASO-210@IFNγ-sEVs in treating psoriasis in a well-established IMQ-induced model, which closely resembles the human disease phenotype according to previously published studies. To establish IMQ-induced psoriasis-like mice, 6-week-old male BALB/c mice were treated with a daily topical dose of 62.5 mg of IMQ cream on the shaved back for six consecutive days. DiD-labeled native sEVs and IFNγ-sEVs were intradermally injected into the mice, and bioluminescent images of IFN-γ stimulated MSC-sEVs taken *in vivo* confirmed a marked accumulation of IFNγ-sEVs on the injured skin of IMQ-induced psoriasis-like mice, as compared to unstimulated native sEVs (Figure 3A). IMQ-induced psoriasis-like mice were injected with phosphate-buffered saline (PBS), IFNγ-sEVs (25 mg/kg), ASO-210 (8 nmol), ASO-210&IFNγ-sEVs (8 nmol +25 mg/kg), or ASO-210@IFNγ-sEVs (8 nmol +25 mg/kg), intradermally 4 times from day 2 to day 5 to test the therapeutic effect (Figure 3B). Halometasone (Hal) was topically applied daily as the positive control (Figure 3B). Simultaneously, toxicological experiments were also performed as biological safety is essential for the clinical application of sEV-loaded drugs. The intradermal administration of all groups of drugs did not cause obvious histopathological damage to the main organs, including the heart, liver, spleen, lung, and kidney in the mice (Figure 3C), demonstrating that IFNγ-sEVs do not cause systemic adverse effects when used for psoriasis therapy. As expected, the PBS control mice developed typical psoriasis-like lesions with evident clinical and pathological changes, together with obvious weight loss during the experiment period (Figures 3D–G). Consistent with the previous report, intradermal administration of ASO-210 ameliorated the pathological phenotype of IMQ-induced psoriasis-like mice (Figures 3D–G). Remarkably, IFNγ-sEVs alleviated the weight loss and disease severity including thickness, erythema, and scales of skin lesions following treatment (Figures 3D,E). In addition, the representative skin photographs and H&E staining also exhibited significant improvement in both clinical manifestations and pathological characteristics on day 7, following the treatment of IFNγ-sEVs (Figures 3F,G). Interestingly, IFNγ-sEVs also reduced the proliferation of keratinocytes as reflected by the decreased levels of Ki67 (Figures 3F,H). Consistent with *in vitro* results, IFNγ-sEVs could cooperate with ASO-210 (ASO-210&IFNγ-sEVs and ASO-210@IFNγ-sEVs) to achieve the inhibitory effect on disease development (Figures 3E–G). Interestingly, ASO-210@IFNγ-sEVs showed the better therapeutic effect than ASO-210&IFNγ-sEVs, indicating that loading into sEVs might improve the therapeutic effect of ASO-210 *in vivo* (Figures 3E–G). These results conclude that the IFNγ-sEVs are effective natural nanovesicles carrying antisense oligonucleotides to protect against psoriasis.

### IFNγ-sEVs Exhaust Th17 Cells and Reduce Inflammatory Cytokine Expression in IMQ-Induced Psoriasis-Like Mice

**FIGURE 4:**
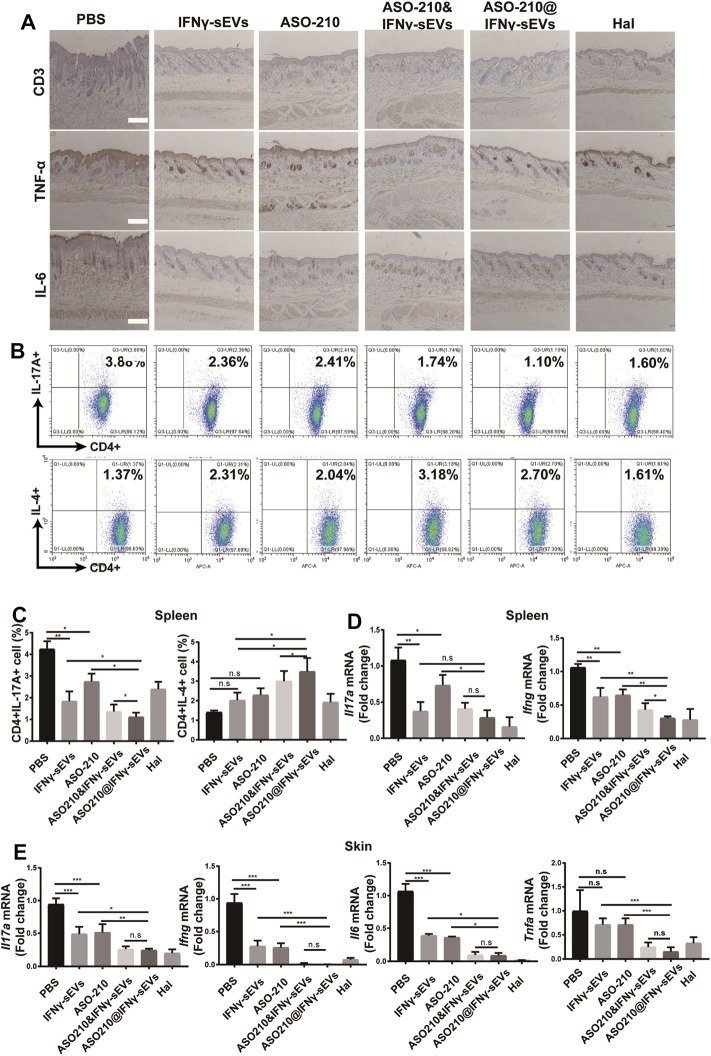
IFNγ-sEVs exhaust Th17 cells and reduce inflammatory cytokine expression in IMQ-induced psoriasis-like mice. **(A)** Immunostaining of CD3, IL-6, TNF-α in skin lesion of the PBS, IFNγ-sEVs, ASO-210, IFNγ-sEVs&ASO-210, and ASO-210@IFNγ-sEVs and Halometasone groups on day 6. Scale bar, 200 μm. **(B,C)** Representative flow cytometry analysis of Th17, and Th2 cells in splenic CD4^+^ T cells from different groups (n = 3). **(D)** qPCR analysis showed mRNA levels of *Il17a* and *Ifng* of the spleen section from different groups on day 6 (n = 3/4). **(E)** qPCR analysis showed mRNA levels of *Il17a*, *Il6*, *Ifng*, and *Tnfa* of the skin section from different groups on day 6 (n = 3/4). Error bar, mean ± SD. n.s represents no significance, *p < 0.05, **p < 0.01, and ***p < 0.001.

**FIGURE 5:**
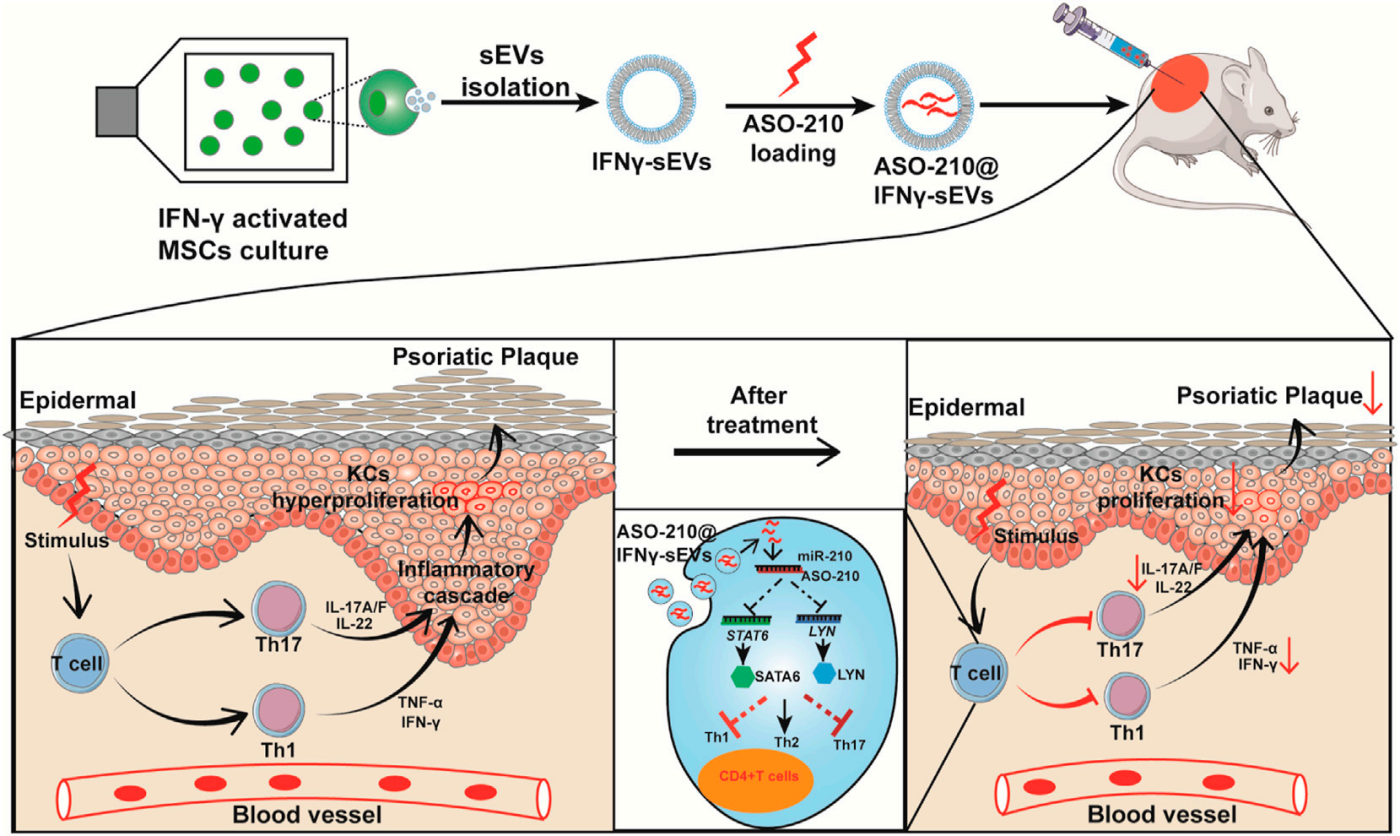
Schematic illustrating the mechanism of ASO-210@IFNγ-sEVs alleviating the pathogenesis of psoriasis. Small extracellular vesicles were purified from the condition medium of huc-MSC stimulated with IFN-γ. ASO-210s were introduced into IFNγ-sEVs by electroporation. Then, we translated the effects of ASO-210@IFNγ-sEVs onto a well-established IMQ-induced model to test their immunosuppressive function *in vivo.* ASO-210@IFNγ-sEVs, administered by intradermal injection, were mostly accumulated in the injured skin, causing a marked reduction of Th17 cell and cytokine secretion such as IL-17A, IFN-γ, TNF-α, and IL-6, and thus inhibit the proliferation of keratinocytes (KCs). These effects contribute to alleviate the formation of psoriatic skin lesions.

In order to further explore the mechanism for the therapeutic effect of IFNγ-sEVs, we carried out the immunohistochemistry analysis of CD3, IL-6, and TNF-α. The results showed that compared with the PBS group, the release of IL-6, TNF-α, and the infiltration of CD3 positive T cells was significantly reduced in all treatment groups, especially in the ASO-210@IFNγ-sEVs group (Figure 4A). Then splenic CD4^+^ T cells were obtained to assess the *in vivo* effect of IFNγ-sEVs and ASO-210 on the immune imbalance of Th cell subsets in a psoriasis-like IMQ mouse model. Notably, both IFNγ-sEVs and ASO-210 increased the percentage of CD4^+^IL-4^+^ T cells (Th2) and decreased the percentage of CD4^+^IL-17A^+^ T cells (Th17) in the spleen of IMQ-treated mice (Figures 4B,C). In addition, IMQ-treated mice injected with IFNγ-sEVs or ASO-210 displayed a prominent reduction of *Il17a*, *Ifng*, *Il6*, and *Tnfa* mRNA in both skin lesions and splenic CD4^+^ T cells (Figures 4D,E). Compared to IFNγ-sEVs, ASO-210, or ASO-210&IFNγ-sEVs, ASO-210@IFNγ-sEVs had more significant effects in preventing the immunopathological changes, which was consistent with milder disease severity in the ASO-210@IFNγ-sEVs group (Figures 4B–E). Together, these results indicated that IFNγ-sEVs exhibited a synergistic immunosuppressive function with ASO-210 in IMQ-induced psoriasis-like mice, while ASO-210@IFNγ-sEVs presented stronger inhibition (Figure 5).

## Discussion

In recent years, MSCs such as adipose tissue-derived MSCs (AT-MSCs) and umbilical cord-derived MSCs (UC-MSCs) are being tested in several clinical trials for psoriasis treatment. However, safety issues, unclear mechanisms of action, and difficulty in reaching the target sites limit its wide application. Interestingly, accumulating evidence suggests that sEVs derived from MSCs display immunoregulatory functions similar to the parent cells. For example, MSC-derived sEVs have been reported to have therapeutic effects in GvHD (Zhou et al., 2020) and autoimmune diseases, including the induction of tissue regeneration (Chen et al., 2008), immunosuppression, and anti-inflammatory properties (Jin-Hee et al., 2018). MSC-sEVs can also be modified to display multiple immunomodulatory molecules on the membrane surface to potentiate their immunosuppressive potential (Tsai et al., 2021). These favorable characteristics underscore the potential of MSC-sEVs as a unique drug delivery vehicle to treat psoriasis. We therefore compared sEVs from a diverse range of MSC sources and found that sEVs derived from human umbilical cord-derived MSCs stimulated by IFN-γ (IFNγ-sEVs) could inhibit T-cell proliferation and differential *in vitro and vivo*. In addition, IFNγ-sEVs reduced the PASI score, back skin thickness, inflammatory cytokine expression, and exhausted inflammatory cells such as Th17 cells in IMQ-induced psoriasis-like mice.

ASO-210 was previously reported to alleviate psoriasis by blocking the immune imbalance and the development of psoriasis-like inflammation, but the instability *in vivo* and low delivery efficiency to the target cell limited their wide application. With small size, lower cytotoxicity and immunogenicity, stability, and specificity to target tissues, sEVs have been identified as potential delivery vehicles of RNA reagents as they can protect the loaded RNAs from RNase and improve delivery efficiency to the target cells. We found IFNγ-sEVs loaded with ASO-210 showed a stronger protective effect against psoriasis than the same amount of ASO-210 given separately with IFNγ-sEVs. A couple of reasons may explain this distinction. First, the accumulation of the drug-loaded sEVs in skin lesions led to the accumulation of drugs at high concentrations in the psoriasis region, which likely contributed to the prominent therapeutic effects of the drug-loaded IFNγ-sEVs. Second, sEVs could protect ASO-210 from hydrolysis by RNase I, likely resulting in the improved stability of the ASO-210 *in vivo*. Therefore, it has been proved that ASO-210 loaded into MSC-sEVs has increased the stability and delivery efficiency to target cells of ASO-210, which promotes the therapeutic effect. Overall, these results reveal that as a natural membrane delivery system, MSC-derived sEVs present themselves as an excellent alternative biological vector with advantages that can also dramatically enhance the therapeutic effects of loaded drugs.

As a summary, we confirmed MSC-sEV function as immunosuppressive agents to prevent the pathology of psoriasis. With lower cytotoxicity and immunogenicity, our study also suggests MSC-sEVs as promising tools for the delivery of nucleotide drugs as MSC-sEVs loaded with a low dose of ASO-210 exhibit a synergistic function in blocking the immune imbalance and alleviating subsequent psoriasis development. We believe that similar strategies can be further explored in the treatment of autoimmune diseases in general.

## Data Availability

The raw data supporting the conclusions of this article will be made available by the authors, without undue reservation.
